# Superior mesenteric artery syndrome in a patient with fibrodysplasia ossificans progressiva

**DOI:** 10.1016/j.bonr.2023.101702

**Published:** 2023-07-20

**Authors:** Tae Young Ahn, Jung Bum Han, Jung Yun Bae, Seung Hun Woo

**Affiliations:** a Department of Orthopedic Surgery, Pusan National University Hospital, Pusan National University School of Medicine, Busan 49241, Republic of Korea; b Department of Orthopedic Surgery, Pusan National University Yangsan Hospital, Pusan National University School of Medicine, Yangsan 50612, Republic of Korea

**Keywords:** Fibrodysplasia ossificans progressive, Superior mesenteric artery syndrome, Weight loss, Gastrointestinal symptoms, Abdominal computed tomography

## Abstract

An 18-year-old boy with fibrodysplasia ossificans progressiva lost weight at an accelerated rate due to gastrointestinal symptoms, resulting in a weight loss of 36 kg in 1 year. His first outpatient abdominal computed tomography (CT) result was unremarkable. Since the patient had biliary vomiting during hospitalization, his CT was reexamined, and the superior mesenteric artery syndrome diagnosis was confirmed. Thus, clinicians must consider superior mesenteric artery syndrome when presented with weight loss.

## Highlights

• An adolescent boy with fibrodysplasia ossificans progressiva lost 36 kg in 1 year.
• A diagnosis of superior mesenteric artery syndrome was made.
• Clinicians must consider superior mesenteric artery syndrome in weight loss cases.

## 1 Introduction

Fibrodysplasia ossificans progressiva is a very rare disease characterized by congenital anomalies of the foot and toe and progressive heterotopic ossification (Kaplan et al., 2005; Pignolo et al., 2019). It is caused by mutations in the activin receptor IA (ACVR1) gene (Shore et al., 2006) and is inherited in an autosomal dominant manner. Most patients have severe disabilities and are sterile; therefore, most patients have normal parents. At birth, fibrodysplasia ossificans progressive patients appear normal except for the deformity of the feet. In addition to hallux valgus, deformities such as absent skeletal structures, malformed epiphyses, ectopic ossification centers, malformed first metatarsals, and phalangeal fusion may also appear (Towler et al., 2020). Flare-ups (swelling and pain in the neck, back, and limbs) occur with or without minor trauma, such as contusions, vigorous exercise, or intramuscular injections. After several days to weeks, ectopic bone is created in the affected area, which reduces the range of motion and eventually hardens the joint (Kaplan et al., 2005). Although the diaphragm, extraocular muscles, and myocardial and smooth muscles are unaffected, other muscles gradually lose their ability to function as they become ossified. Thoracic insufficiency syndrome may occur due to scoliosis and ossification around the rib cage, and patients may die of respiratory disease. There are cases of death due to malnutrition caused by restriction of oral movement (Kaplan et al., 2010).

Gastrointestinal symptoms have been reported to occur in approximately 28 % of patients with fibrodysplasia ossificans progressiva. Abdominal pain is the most common, and symptoms such as reflux, nausea, difficulty swallowing, decreased appetite, constipation, severe diarrhea, digestive disorders, and intermittent vomiting have been reported. However, the cause is mostly unknown (Mantick et al., 2018). Superior mesenteric artery (SMA) syndrome is a rare and potentially life-threatening condition characterized by bowel obstruction resulting from the mechanical compression of the third portion of the duodenum by the superior mesenteric artery, and common causes include low BMI and scoliosis surgery (Lam et al., 2014).

In the present case, weight loss started due to gastrointestinal symptoms commonly associated with fibrodysplasia ossificans progressiva, and superior mesenteric artery syndrome occurred due to weight loss, further exacerbating weight loss.

## 2 Case

### 2.1 Case presentation

**Fig. 1:**
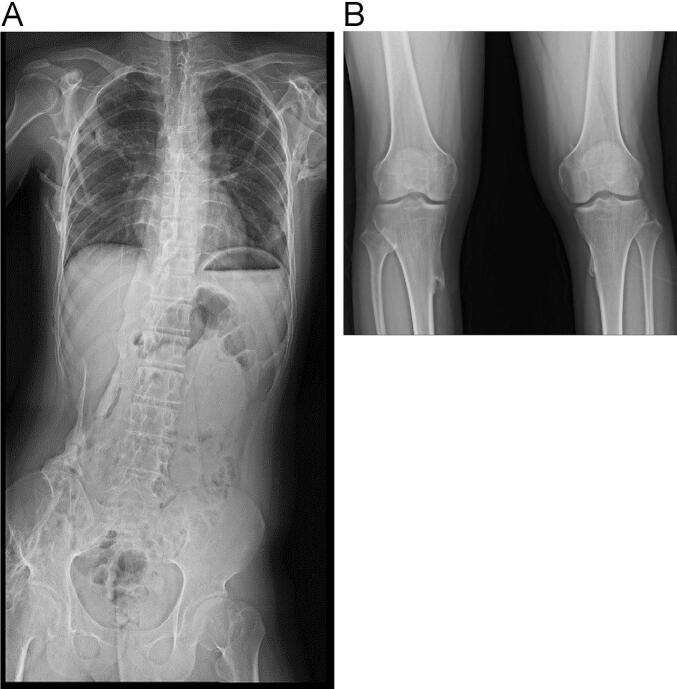
(A) Chest x-ray taken when the patient was in early adolescence. Heterotopic ossification was present in both shoulders and the chest, spine, and ilium. (B) Bony spur present at both proximal tibias.

**Fig. 2:**
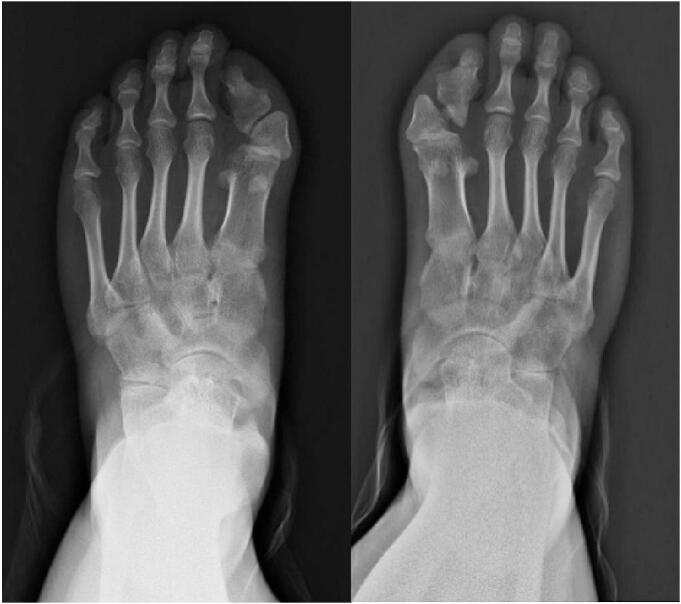
Congenital malformations of the great toes. Slanting of the metatarsophalangeal joint indicating hallux valgus.

The patient was an 18-year-old boy with a diagnosis of fibrodysplasia ossificans progressiva. The genetic diagnostic test was conducted at another hospital approximately 8 years prior, and the exact method is unknown to us. He had a point mutation, c.617G > A; p.R206H, in the *ACVR1* gene. Osteochondroma-like bony spurs and heterotopic ossification were observed on the chest wall, pelvis, shoulder, and knee (Fig. 1). He also showed hallux valgus deformity (Fig. 2). In middle childhood, he had a large mass in his right chest wall, which was resected in another hospital before he was diagnosed with FOP. Six years later, he was admitted to the pediatric orthopedic surgery department due to a flare-up in the region above the left shoulder; thus, intravenous steroids were administered for 4 days. Flare-ups occurred four times, and he was similarly admitted to the pediatric orthopedic surgery department and received steroid treatment.

In late adolescence, the patient suffered continuous weight loss for 6 months. Two years prior, his weight was over 80 kg and had decreased from 58 to 44 kg in 6 months. In addition to weight loss, his oral intake also decreased. The patient ate only one meal daily, which was 40 % of their usual intake. There was no problem with the temporomandibular joint; thus, he could open his mouth and had no difficulty speaking or eating. He also complained of abdominal pain, decreased appetite, and vomiting; these symptoms existed intermittently before the weight loss. During these 6 months of rapid weight loss, there was one occurrence of syncope. Immediately after the syncope, consciousness was recovered, and no seizure occurred.

This study was approved by the Pusan National University Yangsan Hospital Institutional Review Board (IRB No. 05-2021-080). The patient provided written informed consent for the publication of this case report.

### 2.2 Investigations

Persistent and severe weight loss was evaluated at an outpatient department of the pediatric gastroenterology clinic. Abdominal computed tomography (CT), chest CT, and upper gastrointestinal endoscopy were performed. The initial reading of CT of the abdomen showed no specific findings other than an enlarged prostate (4.7 × 3.7 cm), a small number of ascites in the pelvis, and multiple external growth osteophytes of the ilium, lumbar vertebrae, and ribs. Chest CT showed no specific findings other than fibrodysplasia ossificans progressiva, and upper gastrointestinal endoscopy showed no specific findings.

The patient was admitted to the pediatric genetic and metabolic disease clinic for multidisciplinary evaluation and cooperative treatment in the gastroenterology, neurology, cardiology, and urology clinics for persistent weight loss and the occurrence of syncope.

Blood tests, such as complete blood count, liver function test, renal function test, electrolyte test, and lipid profile, were performed as initial tests after hospitalization, and there were no specific findings. Electroencephalogram and brain magnetic resonance imaging, an echocardiogram, a 24-h Holter test, a tilt table test, and a three-position blood pressure test were also performed and were unremarkable. The day after admission, the patient had green biliary vomiting.

**Fig. 3:**
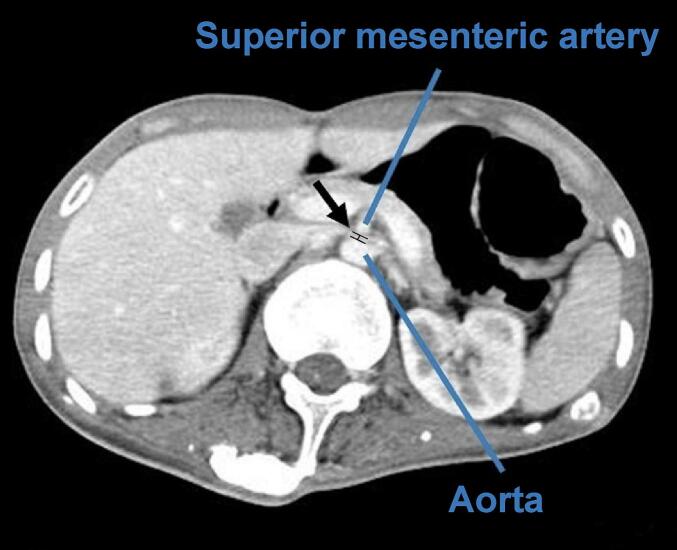
Abdominal CT showing extrinsic compression of the third part of the duodenum between the aorta and superior mesenteric artery. Arrow: Aortomesenteric distance measuring at 4.3 mm (normal value, >10 mm).

We suspected superior mesenteric artery syndrome among several possible causes based on the patient's persistent weight loss, a very thin physique with a body mass index (BMI) of 15.5 kg/m^2^, indigestion, decreased oral intake, and biliary vomiting. Therefore, the previously performed abdominal CT was re-read, and the third part of the duodenum was discovered to be compressed from the outside between the aorta and the superior mesenteric artery. The distance between the aorta and the superior mesenteric artery was 4.3 mm (Fig. 3). Therefore, the diagnosis of superior mesenteric artery syndrome was confirmed.

### 2.3 Treatment

In addition to peripheral complete intravenous nutrition, which was performed during hospitalization for weight gain, the patient was given an oral intake of liquid nutrients. The patient did not experience additional vomiting during the hospitalization period, and oral intake also improved daily.

### 2.4 Outcome and follow-up

The patient recorded a weight of 46.3 kg (BMI = 16.6 kg/m^2^) within 1 week of hospitalization, a 3 kg increase compared to 43.3 kg (BMI = 15.5 kg/m^2^) upon admission. Based on these improvements, the patient was discharged after 9 days of hospitalization. There were no specific findings in the outpatient follow-up. After 10 months of discharge, the weight increased to 50 kg (BMI = 17.9 kg/m^2^).

## 3 Discussion

Herein, a patient previously diagnosed with fibrodysplasia ossificans progressiva lost weight due to persistent abdominal pain, decreased appetite, and vomiting, leading to the diagnosis of superior mesenteric artery syndrome.

Superior mesenteric artery syndrome is a rare disease in which the third part of the duodenum is compressed by the aorta and superior mesenteric artery, resulting in duodenal obstruction. The triggers of superior mesenteric artery syndrome can be classified into three categories: weight loss, external or intra-abdominal pressure, and increased mesenteric tension (Welsch et al., 2007). The clinical course can be divided into the acute and chronic types (Kaiser et al., 1960). In the acute type, epigastric pain and biliary vomiting repeatedly occur due to trauma, surgery, or burns, accompanied by weight loss. In the chronic type, indigestion, nausea, vomiting, and intermittent biliary vomiting are the most common symptoms for several months to years and are accompanied by a low BMI. Unal et al. (2005) found that an angle between the aorta and the mesenteric artery on CT of <22 degrees (sensitivity, 42.8 %; specificity, 100 %) and a distance of <8 mm (sensitivity 100 %, specificity 100 %), and at least one symptom of superior mesenteric artery syndrome could confirm the diagnosis of superior mesenteric artery syndrome. Treatment of superior mesenteric artery syndrome is first started with conservative treatment, and if unsuccessful, surgical treatment is considered. However, in the case of patients with fibrodysplasia ossificans progressiva, surgical or invasive procedures can potentially trigger flare-ups, so they should only be performed when absolutely necessary. In conservative treatment, gaining weight through peripheral complete intravenous nutrition or a nasal jejunum tube is important. A nasogastric tube is inserted for decompression of the duodenum or stomach. For symptom relief, the patient is placed in a prone or left lateral position (Dietz et al., 2000). Although this disease is difficult to diagnose due to its infrequency and diverse symptoms, it is important to consider the diagnosis of superior mesenteric artery syndrome in clinical practice (Kim and Lee, 2013).

The causative factor of this case is weight loss due to the continuation of gastrointestinal symptoms, and it is a chronic clinical course. Superior mesenteric artery syndrome was suspected based on persistent weight loss, gastrointestinal symptoms, and biliary vomiting. Abdominal CT showed duodenal compression and the aorta-superior mesenteric artery distance of 4.3 mm, which was less than the normal 10 mm, which led to the diagnosis of superior mesenteric artery syndrome.

Although superior mesenteric artery syndrome is infrequent and diverse in symptoms, fibrodysplasia ossificans progressiva is also rare, non-specific, and accompanied by various gastrointestinal symptoms. However, patients with fibrodysplasia ossificans progressiva show various gastrointestinal symptoms, such as reflux, nausea, difficulty swallowing, decreased appetite, constipation, severe diarrhea, digestive disorders, and intermittent vomiting, and in most cases, the cause is unknown. We believe that some of these cases may be due to superior mesenteric artery syndrome. In the current case, indigestion, anorexia, and constipation were present even before weight loss worsened; thus, it was difficult to diagnose. In patients with fibrodysplasia ossificans progressiva, there may be weight loss due to various causes, which may also cause superior mesenteric artery syndrome. Therefore, clinical suspicion of superior mesenteric artery syndrome is very important.

Without known cause, patients with fibrodysplasia ossificans progressiva present varied gastrointestinal symptoms, such as reflux, nausea, dysphagia, decreased appetite, constipation, diarrhea, digestive disorder, and intermittent vomiting. Also, although superior mesenteric artery syndrome is difficult to diagnose due to its rare frequency and diverse symptoms, suspicion of it in clinical practice is the key to early diagnosis and appropriate treatment. Lastly, clinicians should consider superior mesenteric artery syndrome if a patient with fibrodysplasia ossificans progressiva has weight loss and biliary vomiting.

## CRediT authorship contribution statement

**Tae Young Ahn:** Conceptualization, Data curation, Formal analysis, Investigation, Methodology, Resources, Supervision, Visualization, Writing – original draft, Writing – review & editing. **Jung Bum Han:** Formal analysis, Investigation, Resources, Writing – original draft. **Jung Yun Bae:** Conceptualization, Data curation, Formal analysis, Investigation, Methodology, Resources, Supervision, Visualization, Writing – original draft, Writing – review & editing. **Seung Hun Woo:** Formal analysis, Investigation, Resources, Writing – original draft.

## Declaration of competing interest

None.

## Data Availability

Data will be made available on request.
